# Expression of Zyxin in Non-Small Cell Lung Cancer—A Preliminary Study

**DOI:** 10.3390/biom12060827

**Published:** 2022-06-13

**Authors:** Aleksandra Partynska, Agnieszka Gomulkiewicz, Aleksandra Piotrowska, Jedrzej Grzegrzolka, Adam Rzechonek, Katarzyna Ratajczak-Wielgomas, Marzenna Podhorska-Okolow, Piotr Dziegiel

**Affiliations:** 1 Division of Histology and Embryology, Department of Human Morphology and Embryology, Faculty of Medicine, Wroclaw Medical University, 50-368 Wroclaw, Poland; agnieszka.gomulkiewicz@umw.edu.pl (A.G.); aleksandra.piotrowska@umw.edu.pl (A.P.); jedrzej.grzegrzolka@umw.edu.pl (J.G.); katarzyna.ratajczak-wielgomas@umw.edu.pl (K.R.-W.); piotr.dziegiel@umw.edu.pl (P.D.); 2 Department of Thoracic Surgery, Wroclaw Medical University, 53-439 Wroclaw, Poland; adam.rzechonek@umw.edu.pl; 3 Division of Ultrastructural Research, Faculty of Medicine, Wroclaw Medical University, 50-368 Wroclaw, Poland; marzenna.podhorska-okolow@umw.edu.pl; 4 Division of Human Biology, Faculty of Physiotherapy, University School of Physical Education in Wroclaw, 51-612 Wroclaw, Poland

**Keywords:** zyxin, non-small cell lung cancer, tumor cells

## Abstract

Background: The potential involvement of zyxin (ZYX) in carcinogenesis has been investigated in many cancer types. However, there are a limited number of studies on the role of ZYX in the progression of non-small cell lung cancer (NSCLC). Since lung cancer is one of the most frequently diagnosed carcinomas, the aim of our study was to determine the localization and expression levels of ZYX in NSCLC and to correlate the results with the clinicopathological data. Materials and Methods: The expression of ZYX was assessed in NSCLC cases and in cell lines representing this tumor type. Levels of ZYX were determined in the clinical material using immunohistochemistry (IHC) and Western Blot. Real-time PCR was used to assess *ZYX* mRNA levels. The expression of ZYX was also checked in NSCLC cell lines using real-time PCR, Western Blot, and immunofluorescence/immunocytochemistry. Results: The results showed lower levels of ZYX in NSCLC cells compared with control tissues. This trend was observed at the protein and mRNA levels. The assays on the NSCLC model also demonstrated lower levels of ZYX in cancer cells compared with control cells. Conclusions: The decreased expression of ZYX in NSCLC may indicate a suppressor role of this protein in NSCLC.

## 1. Introduction

Lung cancer is one of the most commonly diagnosed malignancies worldwide [1] with a high incidence and high mortality rates [1,2]. Therefore, it is one of the most important therapeutic targets. Lung cancer is divided into two subtypes, i.e., non-small cell lung carcinoma (NSCLC) and small cell lung carcinoma (SCLC) [2]. NSCLC accounts for approximately 85% of all new cases of lung cancer [2,3]. NSCLC includes adenocarcinomas (ACs), squamous cell carcinomas (SCCs), and large cell carcinomas (LCCs) [2], of which ACs and SCCs are the most prevalent [3]. Risk factors for lung cancer include long-term smoking, exposure to trace metals, asbestos, and genetic predispositions [3,4].

There is a growing need to search for factors that may be crucial in the development of lung cancer. One of the factors under consideration is zyxin (ZYX), a LIM domain protein commonly known as a component of focal adhesions and stress fibers [5]. ZYX is involved in actin polymerization induced by mechanical stress in structures such as focal adhesions and stress fibers [6], thus enabling remodeling and repair of stress fibers [5]. It has been shown that ZYX undergoes translocation from focal adhesions/cytoplasm to the cell nucleus under the influence of various factors (e.g., mechanical forces, UV, epidermal growth factor, retinoic acid) [7,8,9,10]. Therefore, its role in regulating gene expression has also been postulated [7,10]. Studies have shown that ZYX can interact with many proteins, such as transcription factor ZNF384 (involved in bone metabolism) [11], transcription factor Hepatocyte Nuclear Factor-1β (HNF-1β) [10], and Cell Cycle and Apoptosis Regulator Protein-1 (CARP-1) [8]. Furthermore, ZYX may play a role in von Willebrand factor (vWF) secretion, apoptosis, and epithelial–mesenchymal transition (EMT) [8,12,13].

Many studies have been conducted to determine the role of ZYX in oncogenesis. Overexpression of ZYX has been demonstrated in breast, colorectal, and hepatocellular carcinomas [14,15,16]. It has also been suggested that ZYX may act as a tumor suppressor protein in prostate and bladder cancers [17]. Moreover, this protein is probably involved in the progression of chronic myeloid leukemia and glioma [18,19]. However, there are only a few studies on the involvement of ZYX in lung cancer progression. Cadinu et al. demonstrated a lower expression of ZYX in the HCC4017 (NSCLC) cell line [20]. Mise et al. described the role of ZYX in migration and adhesion of lung cancer cells [21]. The same authors also presented decreased ZYX levels in a mouse model of lung cancer [21]. Other studies aimed to verify ZYX levels in serum exosomes [22] and in plasma [23] of NSCLC patients.

Considering the above, the aim of this study was to verify the location and intensity of ZYX expression in NSCLC and to compare the results with the clinicopathological data.

## 2. Materials and Methods

### 2.1. Patients and Tissue Material

The tissue material was obtained during pulmonary parenchymal resection or lobectomy in patients with NSCLC at the Department of Thoracic Surgery, Wroclaw Medical University between 2007 and 2017. Written informed consent was obtained for the use of clinical material for research. The clinicopathological characteristics of all patients are shown in Table 1. Immunohistochemistry (IHC) reactions were performed on 399 formalin-fixed and paraffin-embedded NSCLC sections (including 169 lung SCCs, 168 lung ACs, and 31 lung LCCs) and 85 non-malignant lung tissue (NMLT) sections. The histological grade (G) of NSCLC cases was determined according to the WHO criteria [24]. TNM Classification of Malignant Tumors eighth edition was used to determine lung cancer stage [25]. Twenty-three frozen NSCLC sections and the corresponding NMLT samples were used for the Western Blot analysis. Real-time PCR was performed on 63 NSCLC specimens and 58 NMLT cases previously fixed in RNAlater solution. Laser microdissection was performed on frozen material including 10 NSCLC sections (5 SCCs and 5 ACs) and 6 NMLTs. The experiments were performed after obtaining the approval of the Bioethics Committee at the Wroclaw Medical University (consent no. KB-483/2018, 6 September 2018 and KB-504/2018, 11 September 2018).

### 2.2. Preparation of Tissue Microarrays (TMAs)

Lung cancer tissue microarrays were prepared from archival formalin-fixed and paraffin-embedded NSCLC and NMLT tissues. Hematoxylin and eosin-stained sections were used to select representative tissue sites using a Pannoramic Midi II Histology scanner (3D Histech, Budapest, Hungary) and the Pannoramic Viewer software version 1.15.4 (3D Histech, Budapest, Hungary). Next, the selected representative cores of 1.5 mm diameter were transferred from the donor block to the target block using the TMA Grand Master instrument (3DHistech, Budapest, Hungary). These TMAs were used for further immunohistochemical reactions.

### 2.3. Immunohistochemistry (IHC)

IHC reactions were performed on 4 µm thick TMA sections placed on Superfrost Plus slides (Menzel Gläser, Braunschweig, Germany). The reactions were performed using the EnVision Flex System (Dako, Glostrup, Denmark). Deparaffinization, rehydration, and antigen retrieval (97 °C, 20 min) were performed in low-pH EnVision FLEX Target Antigen Retrieval Solution (pH = 6) using the PT Link (Dako, Glostrup, Denmark). Dako Autostainer Link48 was used for performing IHC reactions (Dako, Glostrup, Denmark). Endogenous peroxidase activity was blocked by a 5 min incubation with EnVision FLEX Peroxidase-Blocking Reagent (Dako, Glostrup, Denmark). The sections were incubated for 20 min with anti-ZYX monoclonal antibody (1:50, 2D1 clone, catalogue no. sc-293448, Santa Cruz Biotechnology, Dallas, TX, USA) followed by EnVision FLEX + MOUSE LINKER for 15 min. Next, the sections were incubated for 20 min with EnVision FLEX/HRP secondary antibody (Dako, Glostrup, Denmark). DAB+ Chromogen (Dako, Glostrup, Denmark) was used to visualize the reaction. Hematoxylin was used to visualize cell nuclei according to the manufacturer’s instructions (Dako, Glostrup, Denmark).

### 2.4. Assessment of IHC Reactions

IHC reactions were assessed using an Olympus BX41 microscope (Olympus Corporation, Tokyo, Japan). The Remmele and Stegner scoring system (Immunoreactive score—IRS) was used to evaluate the cytoplasmic reaction in cancer cells [26]. This scale is related to reaction intensity (0 points—no reaction; 1 point—weak reaction; 2 points—moderate intensity; 3 points—intense reaction) and the percentage of positive cells (0 points—no positive cells, 1 point—≤10% of positive cells; 2 points—11–50% positive cells; 3 points—51–80% of positive cells; 4 points—>80% of positive cells) [26]. The value of the multiplied components represents the number of points, ranging from 0 to 12. The nuclear reaction was assessed using the scale given in Table 2. The scale includes the percentage of tumor cells in which the nuclear reaction occurred [27].

### 2.5. Cell Lines

Two human NSCLC cell lines, i.e., NCI-H1703 (lung SCC) and NCI-H522 (lung AC) were used (ATCC, Manassas, VA, USA). The IMR-90 normal human lung fibroblast cell line (ATCC, Manassas, VA, USA) was used as the control. NCI-H1703 and NCI-H522 cell lines were cultured in RPMI-1640 medium (Gibco, Grand Island, New York, NY, USA) enriched with 10% FBS (Sigma-Aldrich, St. Louis, MO, USA), 2 mM L-glutamine and antibiotics (Gibco, Grand Island, New York, NY, USA). EMEM medium (Lonza, Basel, Switzerland) supplemented with 10% FBS (Sigma-Aldrich, St. Louis, MO, USA), 1xNEAA (Sigma-Aldrich, St. Louis, MO, USA), sodium pyruvate (Sigma-Aldrich, St. Louis, MO, USA), and 2 mM L-glutamine and antibiotics (Sigma-Aldrich, St. Louis, MO, USA) was used to grow IMR-90 cells. Cells were grown in an incubator at 37 °C with 5% CO_2_.

### 2.6. Immunocytochemistry (ICC)

After 24 h growth, cells were fixed in 4% formaldehyde for 12 min at room temperature (RT) and permeabilized in 0.2% Triton X-100 in PBS for 10 min. The slides were incubated for 5 min with EnVision FLEX Peroxidase-Blocking Reagent (Dako, Glostrup, Denmark). After an hour incubation with anti-ZYX antibody (1:100, 2D1 clone, catalogue no. sc-293448, Santa Cruz Biotechnology, Dallas, TX, USA), the slides were incubated with EnVision FLEX/HRP secondary antibody. DAB+ Chromogen was used for reaction visualization (Dako, Glostrup, Denmark). Hematoxylin was used to stain cell nuclei according to the manufacturer’s instructions (Dako, Glostrup, Denmark).

### 2.7. Immunofluorescence (IF)

Cells were seeded into 8-well Millicell EZ slides (Merck Millipore, Kenilworth, NJ, USA). These cells were left to grow for 24 h and fixed with 4% formaldehyde for 12 min at RT. Permeabilization was performed with 0.2% Triton X-100 in PBS for 10 min. After blocking with 3% BSA in PBST for 45 min, cells were incubated with anti-ZYX antibody (1:100, 2D1 clone, catalogue no. sc-293448, Santa Cruz Biotechnology, Dallas, USA) for 1 h at RT. A secondary anti-mouse antibody conjugated to Alexa-Fluor 488 (1:1000, catalogue no. ab15013, Abcam, Cambridge, UK) was added for 1 h at RT. This was followed by incubation with DAPI (Thermo Fisher Scientific, Waltham, MA, USA) to stain cell nuclei. After washing, the cells were embedded in ProLong Diamond Antifade Reagent (Life Technologies, Carlsbad, CA, USA). Detection and assessment of ZYX expression levels were performed using an Olympus FV3000 confocal microscope (Olympus Corporation, Tokyo, Japan) and CellSense software version 3.2 (Olympus Corporation, Tokyo, Japan).

### 2.8. Western Blot

To isolate proteins from the tissue material, lysis was performed in T-PER Tissue Protein Extraction Reagent, Halt Protease Inhibitor Cocktail (catalogue no. 78510 and 78430, respectively, Thermo Fisher Scientific, Waltham, MA, USA) and 0.66 mM PMSF using TissueLyser LT (Qiagen, Hilden, Germany). The samples were incubated for 30 min at 4 °C and centrifuged (12,000× *g*, 15 min, 4 °C) to collect the supernatant. The cells were lysed using the RIPA buffer (50 mM Tris-HCl pH 8.0, 150 mM NaCl, 0.1% SDS, 1% Igepal CA-630, 0.5% sodium deoxycholate) supplemented with 0.5 mM PMSF and the Halt Protease Inhibitor Cocktail (catalogue no. 78430, Thermo Fisher Scientific, Waltham, USA). After 20-min incubation on ice, the samples were centrifuged (12,000× *g*, 10 min, 4 °C) to collect the supernatant. Protein concentration was measured using the Pierce BCA Protein Assay Kit (catalogue no. 23227, Thermo Fisher Scientific, Waltham, MA, USA). Identical amounts of protein (30 µg per lane) were resuspended in 4× loading buffer (250 mM Tris pH = 6.8, 40% glycerol, 20% (*v*/*v*) β-mercaptoethanol, 0.33 mg/mL bromophenol blue, 8% SDS), denatured for 10 min at 96 °C, and subjected to SDS-PAGE. The proteins were transferred to a PVDF membrane (Immobilon-P; catalogue no. IPVH00005, Merck Millipore, Kenilworth, NJ, USA). The membrane was blocked with 5% skimmed milk (catalogue no. 170-6404, Bio-Rad, Marnes-la-Coquette, France) in 0.1% TBST for 1 h at RT. The membrane was incubated overnight at 4 °C with anti-ZYX antibody (1:200, 2D1 clone, catalogue no. sc-293448, Santa Cruz Biotechnology, Dallas, TX, USA) diluted in 1% milk in 0.1% TBST. The membrane was washed three times and incubated with the HRP-conjugated anti-mouse secondary antibody (AffiniPure Donkey Anti-Mouse IgG (H + L), catalogue no. 715-035-150, Jackson ImmunoResearch, Ely, Cambridgeshire, UK) at a dilution of 1:3000 for 1 h at RT.

After washing, detection was performed with the Immobilon Classico Western HRP Substrate (catalogue no. WBLUC0500, Merck Millipore, Kenilworth, NJ, USA). Densitometric measurements were performed and analyzed using the ChemiDoc MP System instrument and Image Lab Software version 5.0 (Bio-Rad, Marnes-la-Coquette, France). β-actin was used as the reference protein.

### 2.9. RNA Isolation, Reverse Transcription-Quantitative Polymerase Chain Reaction (RT-qPCR)

Total RNA from NSCLC, NMLT, and cell line sections was isolated using the RNeasy Mini Kit (catalogue no. 74104, Qiagen, Hilden, Germany) according to the manufacturer’s instructions. To remove genomic DNA, the samples were digested using the RNase-Free DNase Set (catalogue no. 79254, Qiagen, Hilden, Germany). Reverse transcription was performed using the High-Capacity cDNA Reverse Transcription kit with the RNase inhibitor (catalogue no. 4374966, Applied Biosystems, Foster City, CA, USA) according to the manufacturer’s instructions. The qPCR was performed using the 7500 Real-Time PCR System instrument and 7500 software v2.0.6 (Applied Biosystems, Foster City, CA, USA). The following Taqman probe and primer sets were used in the reactions: *ZYX* (Hs00170299_m1, Applied Biosystems, Foster City, CA, USA) and *ACTB* (Hs99999903_m1, Applied Biosystems, Foster City, CA, USA). Real-time PCR reaction conditions were as follows: polymerase activation at 50 °C for 2 min, initial denaturation at 95 °C for 10 min, denaturation at 95 °C for 15 sec, annealing and extension at 60 °C for 1 min for 45 cycles. β-actin (ACTB) was used as the reference gene. Changes in gene expression were determined using the ΔΔCt method [28]. Reactions were performed in triplicate.

### 2.10. Laser Capture Microdissection (LCM) and Reverse Transcription-Quantitative Polymerase Chain Reaction (RT-qPCR)

Laser capture microdissection (LCM) was performed on NSCLC (SCC and AC) and NMLT specimens. Tumor cells and non-malignant lung cell (NMLC) samples were harvested separately to compare *ZYX* mRNA expression. A Leica CM1950 cryostat (Leica Microsystems, Wetzlar, Germany) was used to slice 10 µm-thick frozen tissue sections that were placed on a polyethylene-terephthalate membrane (catalogue no. 50102, MMI, Glattbrugg, Switzerland). LCM was performed using the MMI CellCut Plus System (MMI, Glattbrugg, Switzerland). RNeasy Micro Kit (catalogue no. 74004, Qiagen, Hilden, Germany) was used to isolate total RNA. The synthesis of cDNA was performed using the QuantiTect Reverse Transcription Kit (catalogue no. 205311, Qiagen, Hilden, Germany). Real-time PCR reactions were performed as described in the section *RNA isolation, reverse transcription–quantitative polymerase chain reaction (RT-qPCR)*.

### 2.11. Statistical Analysis

The results were statistically analyzed using Prism 5.0 (GraphPad, San Diego, CA, USA) and Statistica 13.1 (StatSoft, Krakow, Poland) software. ANOVA with post-hoc Bonferroni’s multiple comparisons test were used to compare ZYX expression in cell lines. The analysis of the results obtained from LCM sections was performed by unpaired t test. Mann–Whitney test or Kruskal–Wallis test with Dunn’s post-hoc multiple comparisons test was used to compare ZYX expression in the groups without Gaussian distribution. The paired t-test or unpaired t-test was used to perform statistical analysis of ZYX protein levels in the tissue material as determined by the Western Blot analysis. The survival analysis was performed using the Kaplan–Meier method and the Mantel–Cox test. Univariate and multivariate analyses were performed with the Cox proportional hazards model. The survival analysis was performed only in cases with the complete clinicopathological data. The results were considered statistically significant at *p* < 0.05.

## 3. Results

### 3.1. Expression of Zyxin in NSCLC Cell Lines and Normal Lung Fibroblasts

**Figure 1:**
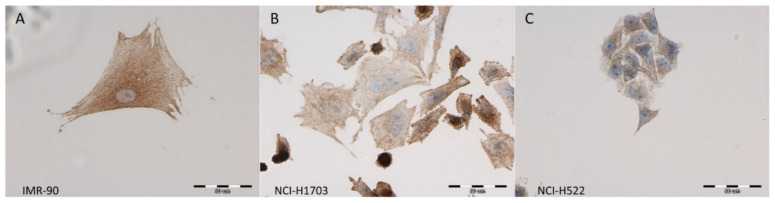
Immunocytochemical (ICC) reactions demonstrating the cytoplasmic localization of ZYX (brown) in the IMR-90 normal lung fibroblast cell line (**A**), NCI-H1703 lung squamous cell carcinoma (SCC) cell line (**B**), and NCI-H522 lung adenocarcinoma (AC) cell line (**C**). Nuclei were counterstained with hematoxylin (blue). Magnification ×400.

**Figure 2:**
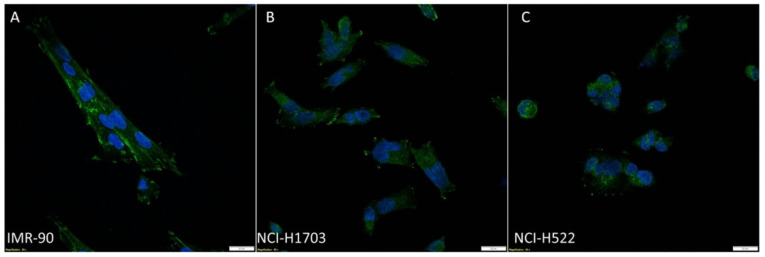
Immunofluorescence (IF) images taken by confocal microscopy showing ZYX expression (green) in the IMR-90 normal lung fibroblast cell line (**A**), NCI-H1703 lung SCC (**B**) and NCI-H522 lung AC (**C**). Nuclei were counterstained with DAPI (blue). Magnification ×600.

**Figure 3:**
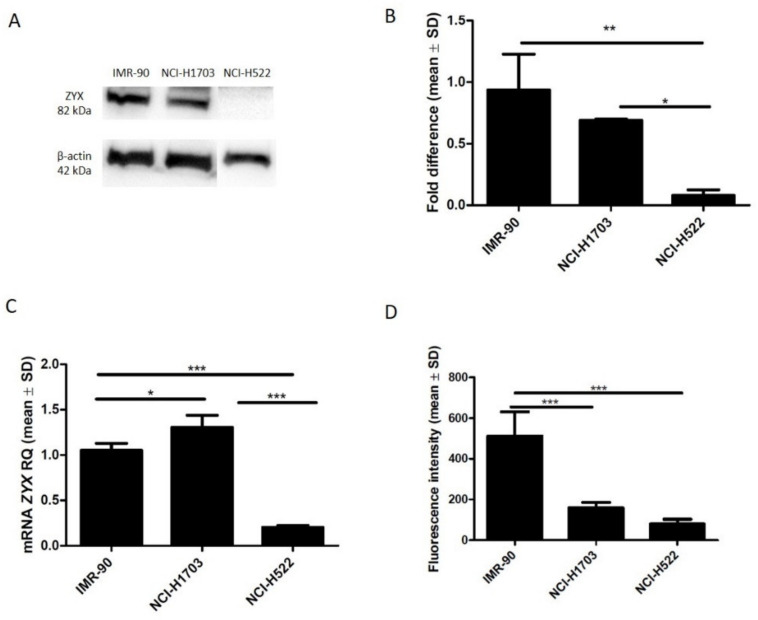
Expression of ZYX in the IMR-90 normal lung fibroblast cell line and in NSCLC cell lines: NCI-H1703 (lung squamous cell carcinoma) and NCI-H522 (lung adenocarcinoma). Western Blot (**A**) and densitometric analysis (**B**) of ZYX protein levels. *ZYX* mRNA expression in the cell lines (**C**). ZYX expression levels determined by measuring fluorescence intensity in the cell lines (**D**). Bonferroni multiple comparisons test (*** *p* < 0.001; ** *p* < 0.01; * *p* < 0.05).

Using ICC and IF, ZYX was detected in the following cell lines: NCI-H1703 (SCC), NCI-H522 (AC), and IMR-90 (normal lung fibroblasts) (Figure 1 and Figure 2). Cytoplasmic and nuclear localization of this protein was observed in all cell lines. Western Blot analysis showed lower levels of ZYX protein in NCI-H1703 and NCI-H522 cell lines compared with control IMR-90 (Figure 3A,B). *ZYX* mRNA expression in NCI-H522 cells was also lower compared with IMR-90 cells (*** *p* < 0.001), while NCI-H1703 cells showed higher *ZYX* mRNA expression compared with control cells (* *p* < 0.05) (Figure 3C). In addition, ZYX expression at mRNA and protein levels was significantly higher in the NCI-H1703 SCC cell line compared with the NCI-H522 AC cell line (*** *p* < 0.001 and * *p* < 0.05; respectively) (Figure 3B,C). Fluorescence intensity measurements showed decreased levels of ZYX in NSCLC cell lines compared with the control line (IMR-90) (Figure 3D).

### 3.2. ZYX Expression in Patients with NSCLC

**Figure 4:**
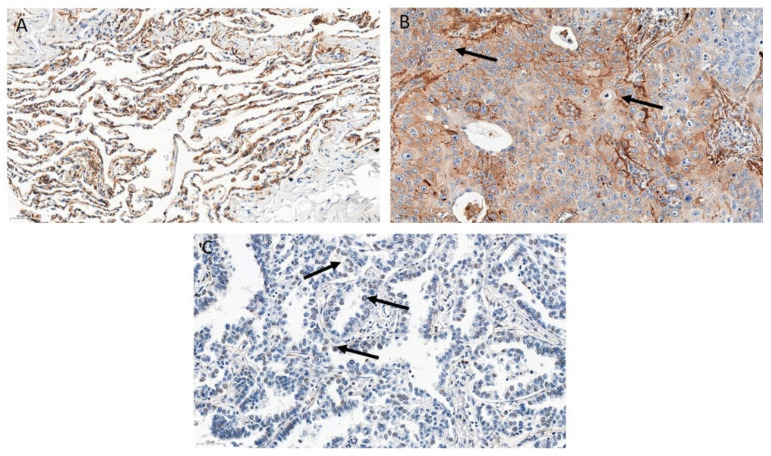
Immunohistochemical (IHC) reactions detecting ZYX protein (brown) performed on non-malignant lung tissue (NMLT) (**A**), lung squamous cell carcinoma (SCC) (**B**), and lung adenocarcinoma (AC) (**C**). Arrows in (**B**) indicate cytoplasmic/membranous localization of ZYX, while arrows in (**C**) show nuclear one. Magnification ×200.

IHC reactions showed the presence of ZYX in the cytoplasm, cell membrane, and in the cell nucleus of tumor cells and NMLT cells. A positive reaction was also found in macrophages in the lung tissue (Figure 4). The representative images of ZYX expression in different lung AC subtypes were also included (Figure S1). The intensity of ZYX expression which was less than or equal to the median in NSCLC cells (IRS ≤ 0.33; nuclear score ≤ 0.5) was defined as “low expression”, while the value of ZYX expression above the median was defined as “high expression”. Cytoplasmic expression of ZYX in tumor cells was found in 207 (51.88%) NSCLC cases. The mean value of cytoplasmic ZYX expression in NSCLC cells was 1.354 ± 1.616 (mean ± SD). There were 201 (50.38%) cases with low cytoplasmic expression of ZYX in NSCLC cells, and 198 (49.62%) cases with high cytoplasmic expression of ZYX. Nuclear expression of ZYX in NSCLC cells was found in 282 (70.68%) cases. The mean value of nuclear expression of ZYX in NSCLC cells was 0.5242 ± 0.4662 (mean ± SD). Low expression of nuclear ZYX in NSCLC cells was present in 236 cases, while high expression was noted in 163 cases.

**Figure 5:**
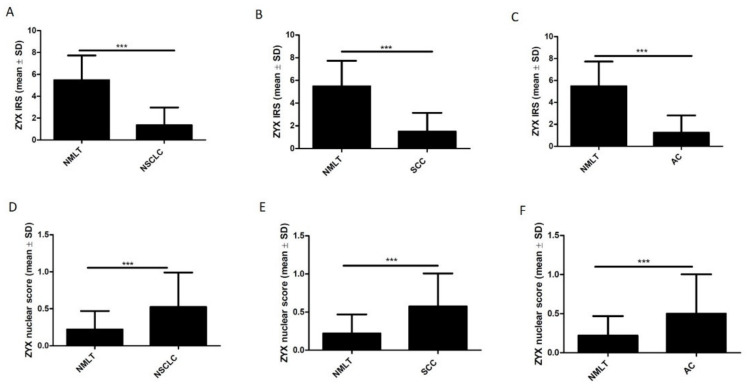
Immunohistochemical (IHC) analysis of ZYX expression in NSCLC cells and in NMLT cells. The graphs showing the intensity of cytoplasmic (IRS) and nuclear (nuclear score) expression of ZYX for the whole NSCLC group (**A**,**D**, respectively), levels of cytoplasmic and nuclear ZYX in lung SCCs (**B**,**E**, respectively), levels of cytoplasmic and nuclear ZYX in lung ACs (**C**,**F**, respectively) compared with NMLT. Mann–Whitney test (*** *p* < 0.001).

Statistical analysis of IHC reactions results showed significantly lower levels of cytoplasmic ZYX in NSCLC cells compared with control tissue (NMLT) (*p* < 0.0001). However, nuclear ZYX levels were increased in tumor cells compared with control tissue (*p* < 0.0001). These findings were observed not only when the total NSCLC group was analyzed, but also when the SCC and AC subtypes were selected (Figure 5).

**Figure 6:**
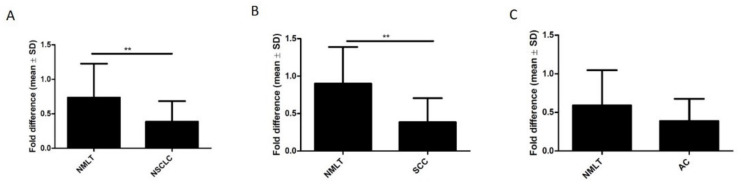
Expression of zyxin in NSCLC (**A**), in the SCC subtype (**B**), and in the AC subtype (**C**) compared with control tissue; Western Blot. Paired t-test (** *p* < 0.01).

**Figure 7:**
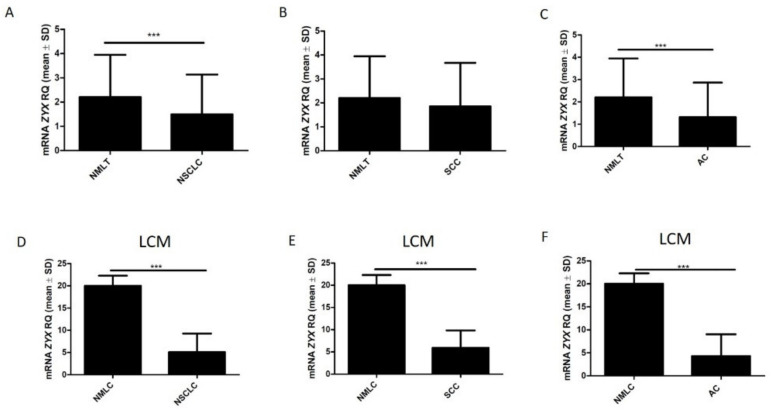
*ZYX* mRNA levels in NSCLC (**A**), lung SCCs (**B**), and lung ACs (**C**) compared with control tissue as determined by real-time PCR. Mann–Whitney test (*** *p* < 0.001). *ZYX* mRNA levels were determined by real-time PCR reactions performed on isolated NSCLC cells (**D**), lung SCC cells (**E**), lung AC cells (**F**), and non-malignant lung cells (NMLCs). Cell isolation was performed by laser capture microdissection (LCM). Unpaired t test (*** *p* < 0.001).

The analysis using Western Blot showed significantly reduced expression of ZYX in NSCLC compared with NMLT samples (*p* = 0.0021). Compared with control tissue, reduced levels of ZYX were also found in lung SCCs (*p* = 0.0075) (Figure 6A,B). Lower levels of ZYX were detected in lung ACs compared with control tissue. However, no statistical significance was reported (*p* = 0.1254) (Figure 6C). *ZYX* mRNA levels were significantly decreased in NSCLC and in lung ACs compared to non-malignant lung tissue (*p* = 0.0005; *p* = 0.0001; respectively) (Figure 7A,C). Real-time PCR performed on laser capture microdissected sections showed lower *ZYX* mRNA expression in tumor cells of the whole NSCLC group (*p* < 0.0001), of the SCC subtype (*p* < 0.0001), and of the AC subtype (*p* < 0.0001) compared with non-malignant lung cells (NMLC) (Figure 7D–F).

**Figure 8:**
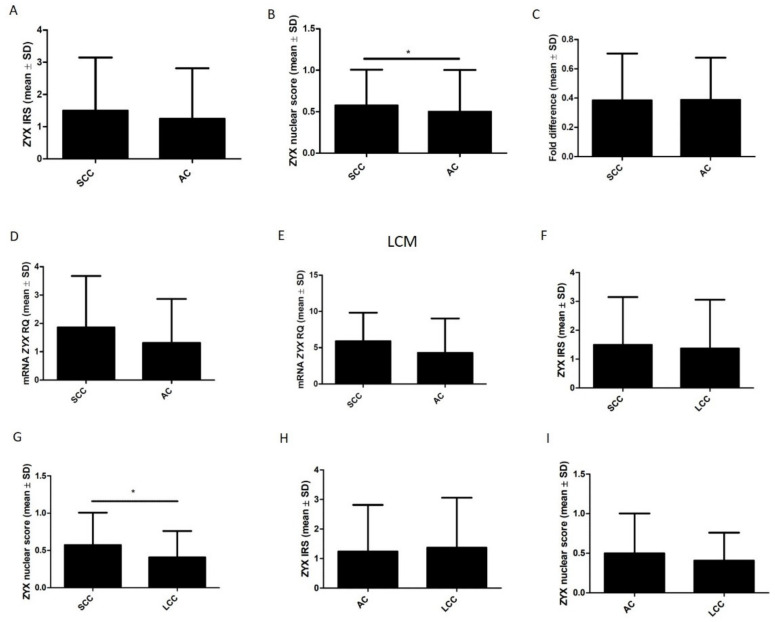
Comparison of zyxin expression in lung SCCs and lung ACs using the following methods: immunohistochemistry (**A**,**B**); Western Blot (**C**); real-time PCR (**D**); and real-time PCR performed on tumor cells isolated using laser capture microdissection (LCM) (**E**). Mann–Whitney test (IHC, RT-qPCR) and unpaired t-test (Western Blot, LCM real-time PCR) (* *p* < 0.05). Comparison of zyxin expression in lung LCCs and other NSCLC subtypes (SCC, AC) with the use of IHC (**F**–**I**). Mann–Whitney test (* *p* < 0.05).

IHC reactions showed higher cytoplasmic and nuclear levels of zyxin in SCC cells than in AC cells (Figure 8A,B). However, statistical significance was observed only in the case of nuclear localization (*p* = 0.0292). Real-time PCR showed higher *ZYX* mRNA levels in the SCC subtype than in the AC subtype (Figure 8D). However, statistical significance was not observed (*p* = 0.2604). Statistical significance was not found when cancer cell isolation was performed using LCM (*p* = 0.5758) (Figure 8E). Western Blot results showed no significant differences in ZYX levels between lung SCCs and lung ACs (*p* = 1.000) (Figure 8C). IHC analysis demonstrated no difference in cytoplasmic ZYX expression between lung SCCs and lung LCCs (*p* = 0.6746) (Figure 8F). Interestingly, higher nuclear ZYX level was detected in SCC than in LCC cells (*p* = 0.0404) (Figure 8G). Higher cytoplasmic ZYX expression in LCC than in AC cells was shown however statistical significance was not observed (*p* = 0.9551) (Figure 8H). Nuclear ZYX level was higher in AC cells than in LCC cells but no statistical difference was noticed (*p* = 0.5319) (Figure 8I).

**Figure 9:**
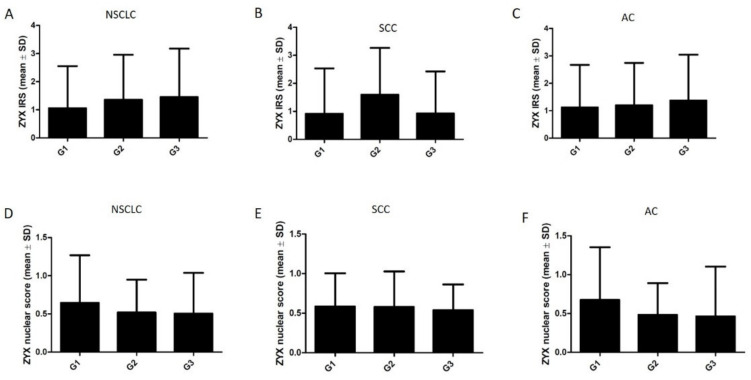
Immunohistochemistry (IRS, nuclear score) of ZYX expression in NSCLC cells in relation to the histological grade. The analysis for NSCLC (**A**,**D**), lung SCC (**B**,**E**), and lung AC (**C**,**F**). Dunn’s multiple comparisons test.

**Figure 10:**
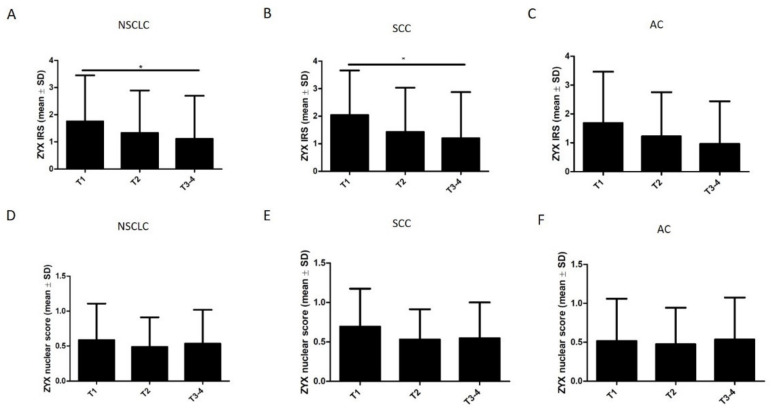
Immunohistochemistry (IRS, nuclear score) of ZYX expression in NSCLC cells in relation to tumor size (pT). Results for NSCLC (**A**,**D**), SCC (**B**,**E**), and AC (**C**,**F**) subtypes. Dunn’s multiple comparisons test (* *p* < 0.05).

**Figure 11:**
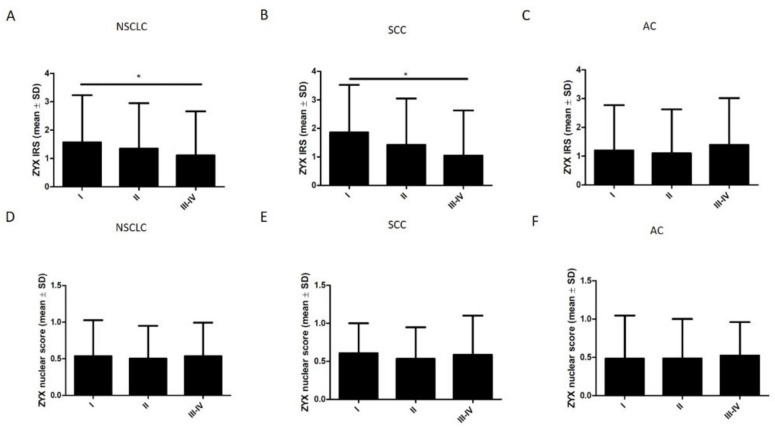
Immunohistochemistry (IRS, nuclear score) of ZYX expression at different clinical stages of cancer. The analysis for the whole NSCLC group (**A**,**D**), SCC (**B**,**E**), and AC (**C**,**F**) subtypes. Dunn’s multiple comparisons test (* *p* < 0.05).

The levels of both cytoplasmic and nuclear ZYX in NSCLC, SCC, and AC cells were not significantly different according to the histological grade (G) (Figure 9). Further analyses showed that the levels of cytoplasmic ZYX in NSCLC cells decreased with increasing tumor size (pT) (Figure 10A). The same relationships were observed when tumor size (pT) was compared with the levels of cytoplasmic ZYX in lung SCC and lung AC cells (Figure 10B,C). Significantly lower levels of cytoplasmic ZYX in NSCLC and SCC cells were demonstrated for T3-4 compared with T1 (* *p* < 0.05, for both). The levels of nuclear expression of ZYX in NSCLC, SCC, and AC cells showed no relationships (Figure 10D–F). The analysis of the relationship between the intensity of ZYX expression and the clinical stage showed that the levels of cytoplasmic ZYX in NSCLC cells decreased with the increasing stage (Figure 11A). The same relationship was also present for cytoplasmic ZYX in SCC cells. Significantly lower levels of cytoplasmic ZYX in NSCLC and SCC cells were demonstrated in Stage III–IV compared with Stage I (* *p* < 0.05, for both) (Figure 11A,B). The levels of cytoplasmic ZYX in AC cells showed no relationship with the clinical stage. For nuclear ZYX expression, no correlation was shown between the clinical stage and its levels in NSCLC, SCC, and AC cells (Figure 11D–F).

### 3.3. Survival Analysis

**Figure 12:**
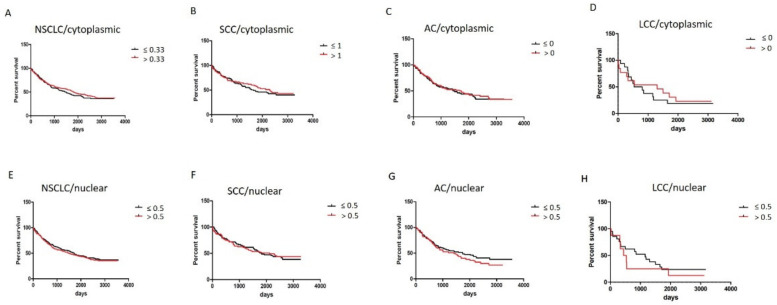
Survival analyses of NSCLC patients in relation to cytoplasmic/nuclear ZYX expression in the cells of NSCLC (**A**,**E**, respectively), SCC (**B**,**F**, respectively), AC (**C**,**G**, respectively), and LCC subtypes (**D**,**H**, respectively). The cut-off point was established in relation to the median value.

Based on IHC, Mantel–Cox tests showed that higher levels of cytoplasmic ZYX in tumor cells were associated with longer overall survival (OS). However, no statistical significance was observed (Figure 12A–D). In terms of nuclear localization, patients with higher ZYX levels had shorter OS except for SCC patients. However, the results were not statistically significant (Figure 12E–H).

**Figure 13:**
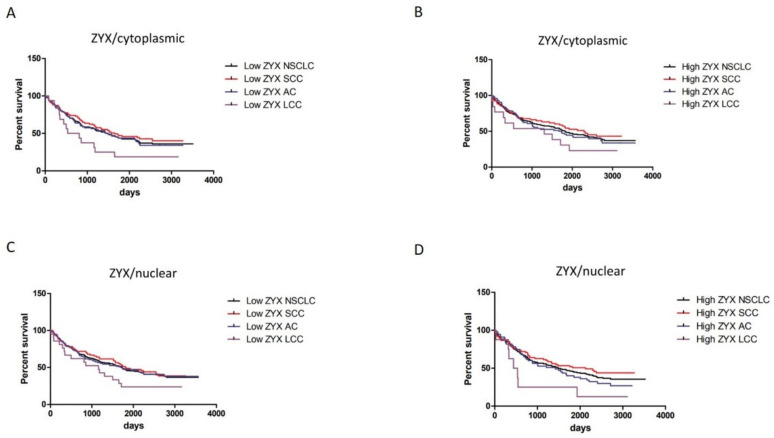
Different cytoplasmic (**A**,**B**) and nuclear (**C**,**D**) ZYX expression statuses in relation to survival of NSCLC (total), SCC, AC, and LCC patients. The cut-off points were set on median values of ZYX expression in NSCLC, SCC, AC, and LCC (IHC).

Analyses of different ZYX expression groups (low/high level) in relation to survivals of patients demonstrated that overall survivals of NSCLC (in total), SCC, and AC patients were similar in each group (Figure 13A–D). The differences were observed in the case of LCC patients who had shorter OS in comparison with NSCLC, SCC, and AC patients.

Univariate analysis showed that OS of NSCLC patients was associated with the clinicopathological factors such as age over 62 years, male sex, higher histological grade (G), larger tumor size (pT), presence of lymph node metastases (pN), and higher clinical tumor stage (Table 3). It was shown that cytoplasmic and nuclear expression of ZYX in NSCLC could not be considered a factor affecting patient survival. For patients with SCC, higher clinical stage, higher histological grade (G) and higher pT affected OS. ZYX expression in SCC did not affect OS (Table 3). Univariate survival analysis of AC patients showed that the male sex, living in urban areas, higher clinical stage, higher pT and the presence of lymph node metastases (pN) were negative factors for survival. Cytoplasmic/nuclear expression of ZYX in AC was not a factor affecting OS (Table 3).

Multivariate survival analysis was performed for all factors that were statistically significantly associated with OS in univariate analyses. Age over 62 years, male sex, higher histological grade (G), tumor size (pT), and higher clinical stage were shown as independent prognostic factors for patients with NSCLC (Table 3). Multivariate survival analysis for patients with SCC showed that histological grade (G) and pT had an independent prognostic effect on survival (Table 3). In turn, multivariate survival analysis for AC patients showed that the male sex, living in urban areas, higher pT, and the presence of lymph node metastases (pN) were independent prognostic factors (Table 3).

## 4. Discussion

Few and controversial papers related to the role of ZYX in NSCLC prompted us to investigate this issue. Moreover, to the best of our knowledge, these studies are among the few in which the assessment of ZYX expression was performed in NSCLC cases using clinical specimens.

There are many reports demonstrating that ZYX may function not only as an oncogenic protein but also as a suppressor protein in the process of carcinogenesis [17,29]. Increased levels of ZYX have been demonstrated in breast and colorectal cancers [14,15], while a suppressor role of this protein has been found in prostate and bladder cancers [17,30,31]. In NSCLC, ZYX probably functions as a suppressor protein, as demonstrated by our results.

By using Western Blot and real-time PCR, we demonstrated that the total level of ZYX in NSCLC cases was significantly decreased compared with normal lung tissue. RT-qPCR results obtained using laser microdissected sections confirmed lower *ZYX* mRNA expression in tumor cells compared with normal cells. IHC reactions also showed lower cytoplasmic ZYX expression in cancer cells compared with normal cells. The decreased levels of ZYX in cancer cells were also demonstrated by studies using an in vitro model of NSCLC cell lines. Our results are consistent with the reports of Mise et al., who demonstrated decreased expression of ZYX in cancer tumors in a mouse model of lung cancer [21]. They also observed that A549 cells with silenced ZYX expression had an increased ability to migrate [21]. This may indirectly suggest that ZYX may inhibit the epithelial-mesenchymal transition (EMT) of lung cancer cells [21]. Cadinu et al. showed lower expression of ZYX in the NSCLC cell line (HCC4017) compared with the control line [20]. Moreover, they also observed significantly lower levels of other cytoskeleton proteins in cancer cells compared with normal cells [20]. These studies suggest that decreased expression of ZYX and cytoskeletal proteins may promote the development of NSCLC. An explanation for this phenomenon may be the disruption of cell adhesion, which enables cell migration and invasion that are closely related to cancer progression.

A suppressor role of ZYX was also reported in prostate cancer [30]. Yu and Luo [30] showed that ZYX could inhibit cell migration and invasion through direct interaction with the protein known as myopodin [17,30]. These results [30] suggest that the impaired interaction of myopodin with ZYX may have a tumor-promoting effect. A similar situation may occur in NSCLC, i.e., impaired interaction of ZYX with other factors may promote the process of carcinogenesis due to reduced levels of ZYX.

In turn, Sanchez-Carbayo et al. showed that low levels of ZYX were associated with higher histological grade and higher clinical stage of bladder cancer [31]. It was suggested that impaired interaction between β-catenin and moesin, E-cadherin, or ZYX could impair the formation of cell adhesion junctions/adherens junctions and thus promote tumor progression [17,31,32]. It seems that similar interactions between ZYX and other proteins may also occur in the development of NSCLC. However, the specific mechanisms have not been described yet.

IHC reactions showed that the levels of ZYX in the cytoplasm were lower in NSCLC cells, while the nuclear expression of ZYX was higher compared with control tissue. An explanation for this phenomenon may be the translocation of ZYX from the cytoplasm to the cell nucleus, previously observed by other researchers [7,9,10,33,34]. The presence of ZYX in the nucleus may be related to its involvement in the regulation of gene expression responsible for the process of cancer transformation [7,10,33].

Moon et al., showed that treatment of SiHa cells with exogenous thymosin β4 resulted in translocation of ZYX to the cell nucleus during the first hours of incubation [34]. After some time, ZYX was translocated back to the cytoplasm [34]. They speculated that under the influence of thymosin β4, ZYX could affect cell migration by regulating actin polymerization and depolymerization. It can also be speculated that ZYX may be a transport molecule for thymosin β4, allowing it to enter the cell nucleus [34]. Thus, it can be suggested that in the case of NSCLC, ZYX may regulate actin polymerization or may function as a transport molecule for other molecules into the nucleus, thus enabling tumor progression.

The presence of ZYX in the cell nucleus may also be related to its interaction with transcription factors and regulation of gene expression that are important in carcinogenesis, including NSCLC. Choi et al. observed that the activation of the transcription factor HNF-1β by ZYX affected the migratory capacity of cells [10]. ZYX has also been shown to interact with the ZNF384 transcription factor (zinc finger protein 384) and it probably functions as a mediator for interactions between ZNF384 and p130CAS in focal adhesions [11]. The role of ZYX in transcriptional regulation was also reported by Degenhardt and Silverstein [33]. Their study [33] showed that ZYX was translocated to the cell nucleus and transcription processes were activated under the influence of the E6 protein of HPV6 [33]. In turn, Youn et al. described the role of ZYX in the regulation of retinoic acid (RA) signaling pathway [9]. Incubation of H1299 NSCLC cells with RA resulted in translocation of ZYX to the nucleus [9]. Further analysis showed the interaction of ZYX with PTOV1 (commonly overexpressed in prostate cancer [35]) and CBP proteins (RA receptor coactivator), resulting in attenuation of the cytotoxic effect of RA [9]. This suggests that ZYX may be responsible for the resistance of cancer cells to therapy. The distribution of ZYX in cells was also reported by Grunewald et al. [36,37]. In their study on ovarian cancer and breast cancer cell lines, they observed that altered expression of LASP-1 protein, which is a focal adhesion molecule [38], resulted in changes in the amount of ZYX in focal adhesions, which affected cell proliferation and migratory abilities [36,37].

Translocation of ZYX to the cell nucleus may also affect the regulation of apoptosis. Ghosh et al. found that mechanical stimulation in the form of stretch resulted in ZYX translocation to the nucleus of vascular smooth muscle cells, which regulated the activity of specific genes [7]. At the same time, silencing of ZYX expression resulted in increased proliferation and inhibition of apoptosis induced by the stretch mechanism and Fas Ligand [7]. Hervy et al. aimed to verify the effect of ZYX on the survival of mouse embryo fibroblasts (MEFs) treated with UV-C [8]. When exposed to UV-C, translocation of ZYX to the nucleus occurred, and increased caspase-3 activity and induction of apoptosis were observed [8]. Further experiments showed that the proapoptotic effect of ZYX was dependent on CARP-1 [8]. The above results confirm that ZYX can promote apoptosis. It is known that apoptosis is one of the processes responsible for the elimination of cancer cells. In our study, the levels of nuclear ZYX in NSCLC cases were significantly higher compared with normal lung tissue, which may indicate that ZYX is translocated to the nucleus to induce apoptosis as a defense mechanism against further tumor progression. However, the expression of nuclear ZYX decreased with the increase in the histological grade of NSCLC, which was mostly found in AC cases. Although these differences were not statistically significant, the trend suggests that this hypothetical defense mechanism was ineffective. Thus, the decrease in ZYX expression, which was observed in our study, may increase the survival of tumor cells and thus promote tumor progression.

The hypothesis of a suppressor role of ZYX in NSCLC development may also be supported by the results of the analysis of the relationship between the level of this protein and tumor size. In our study, we demonstrated that cytoplasmic ZYX levels in all NSCLC cases and in the SCC subtype (which was analyzed separately) decreased with increasing tumor size. We further found that the levels of cytoplasmic ZYX in NSCLC and SCC cells decreased progressively with increasing clinical stage. The nuclear ZYX levels showed no relationship with tumor size or clinical stage. Additionally, we did not observe significant relationships between ZYX expression and histological grade (G). Although nuclear ZYX levels in NSCLC cases analyzed in total and separately for SCC and AC cases decreased slightly with increasing histological grade (G), our results were not statistically significant. The absence of correlations between ZYX levels and some clinicopathological factors may be explained by different mechanisms that cause increase or decrease in ZYX expression. Due to the lack of similar studies in NSCLC, it is not possible to compare our findings. However, they are partly in line with the observations of Sanchez-Carbayo et al., who demonstrated a correlation between low ZYX levels and higher histological grade and higher clinical stage of bladder cancer [31].

The hypothesis of a suppressive role of ZYX can be supported by survival analysis, which showed that higher levels of cytoplasmic ZYX in cancer cells were associated with longer OS. In turn, higher levels of nuclear ZYX correlated negatively with the duration of OS (except for SCC), which is contrary to the results of Hervy et al. [8]. Survival analysis using Cox proportional hazards model showed that cytoplasmic and nuclear ZYX in NSCLC, SCC and AC cells could not be considered to be independent prognostic factors for OS.

Investigation showed that in different ZYX expression groups, overall survivals of NSCLC, SCC, and AC patients did not differ dramatically from one another in each group. However, the differences were noticed for survival curves of LCC patients. The explanation for this phenomenon might be the fact lung LCC is described as a cancer with poor prognosis [39].

Further analysis showed that the levels of both cytoplasmic and nuclear ZYX were higher in SCC cells compared with AC cells. A similar difference was observed in *ZYX* mRNA levels in SCC and AC cases and the corresponding cell lines. This suggests that ZYX expression is regulated differently in the two NSCLC subtypes. This differentiation may be due to the distinct tumor microenvironment that determines tumor properties and may influence the nature of a particular subtype of NSCLC [3]. The structure of the extracellular matrix (ECM) probably affects the expression of ZYX. Fibronectin has been shown to be part of the ECM in desmoplastic pulmonary AC, whereas keratin has been found in lung SCC [3]. Therefore, variation in ECM composition may account for the different expression of ZYX in lung SCC and AC cells. The comparison of cytoplasmic and nuclear ZYX expression between LCC and other NSCLC subtypes demonstrated different levels of this protein. Significantly higher nuclear ZYX levels were observed in SCC than in LCC cells. These facts may also be explained by different ECM composition.

The experiment was carried out on TMAs which do not constitute the whole tissue section. The next limitation is the use of only two NSCLC cell lines. Nevertheless, these cell lines represent the most common types of NSCLC, i.e., lung squamous cell carcinoma (NCI-H1703) and lung adenocarcinoma (NCI-H522). The final conclusions were stated based on the overall and cytoplasmic ZYX expression because the overall level was detected as decreased in NSCLC. However, the cytoplasmic and nuclear levels of ZYX were analyzed in relation to clinicopathological data and discussed. The roles of cytoplasmic and nuclear localizations of ZYX were not investigated with experiments.

Previous studies suggested that ZYX could act as both a promoter and suppressor protein in the process of tumor transformation, depending on the type of cancer. Our results support the conclusion that a decrease in ZYX expression may promote the formation of NSCLC. However, the role of decreased ZYX expression in NSCLC is still not completely explained.

## Figures and Tables

**Table 1:** Clinicopathological characteristics of patients with non-small cell lung cancer (NSCLC).

Clinical Feature	NSCLC		SCC		AC		LCC	
	*n* = 399	%	*n* = 169	%	*n* = 168	%	*n* = 31	%
Age								
≤62	212	53.13%	≤64 89	52.66%	≤61 90	53.57%	≤62 16	51.61%
>62	187	46.87%	>64 80	47.34%	>61 78	46.43%	>62 15	48.39%
Sex								
Female	116	29.07%	32	18.93%	65	38.69%	6	19.35%
Male	283	70.93%	137	81.07%	103	61.31%	25	80.65%
Histological grade								
G1	24	6.02%	4	2.37%	19	11.31%	1	3.23%
G2	293	73.43%	143	84.62%	109	64.88%	24	77.42%
G3	66	16.54%	22	13.02%	38	22.62%	6	19.35%
No data	16	4.01%	0	0.00%	2	1.19%	0	0.00%
Tumor size								
T1	88	22.06%	39	23.08%	33	19.64%	8	25.81%
T2	186	46.62%	76	44.97%	86	51.19%	13	41.94%
T3	79	19.80%	37	21.89%	31	18.45%	6	19.35%
T4	46	11.53%	17	10.06%	18	10.71%	4	12.90%
Lymph node metastases								
N0	260	65.16%	110	65.09%	108	64.29%	19	61.29%
N1	70	17.54%	40	23.67%	22	13.10%	4	12.90%
N2	69	17.29%	19	11.24%	38	22.62%	8	25.81%
Distant metastases								
M0	395	99.00%	169	100.00%	165	98.21%	30	96.77%
M1	4	1.00%	0	0.00%	3	1.79%	1	3.23%
Clinical stage								
I	145	36.34%	64	37.87%	62	36.90%	7	22.58%
II	130	32.58%	64	37.87%	46	27.38%	12	38.71%
III	120	30.08%	41	24.26%	57	33.93%	11	35.48%
IV	4	1.00%	0	0.00%	3	1.79%	1	3.23%
Smoking								
Smokers	336	84.21%	157	92.90%	128	76.19%	29	93.55%
Non-smokers	63	15.79%	12	7.10%	40	23.81%	2	6.45%
Living in urban areas								
Yes	31	7.77%	16	9.47%	10	5.95%	1	3.23%
No	368	92.23%	153	90.53%	158	94.05%	30	96.77%
Death								
Yes	244	61.15%	92	54.44%	108	64.29%	24	77.42%
No	147	36.84%	74	43.79%	59	35.12%	6	19.35%
No data	8	2.01%	3	1.78%	1	0.60%	1	3.23%

**Table 2:** Scoring system for nuclear reaction intensity (modified according to [27]).

Points	Percentage of Cells with Positive Nuclear Reaction
0	0%
1	≤10%
2	11–25%
3	26–50%
4	>50%

**Table 3:** Survival analysis of patients with NSCLC (A), SCC (B), and AC (C). The analyses were performed using Cox proportional hazards model.

A						
		Overall Survival				
		NSCLC				
Univariate				Multivariate		
Clinical Feature	*p* Value	HR	Confidence Interval 95% (HR)	*p* Value	HR	Confidence Interval 95% (HR)
Age						
≤62 vs. >62	**0.005911**	**1.459308**	**1.114989–1.909957**	**0.001879**	**1.540898**	**1.173239–2.023770**
Sex						
Female vs. male	**0.000262**	**1.820726**	**1.319828–2.511725**	**0.000222**	**1.845819**	**1.333147–2.555644**
Smoking						
No vs. Yes	0.164272	1.325817	0.891007–1.972812			
Living in						
urban areas						
No vs. Yes	0.053255	1.575759	0.993642–2.498906			
Clinical stage						
I–II vs. III–IV	**0.000000**	**2.351361**	**1.782315–3.102088**	**0.009199**	**1.702469**	**1.140750–2.540783**
Histological grade						
G1–G2 vs. G3	**0.035460**	**1.441683**	**1.025203–2.027353**	**0.011715**	**1.564191**	**1.104626–2.214952**
pT						
pT1-pT2 vs.						
pT3-pT4	**0.000000**	**2.161526**	**1.639607–2.849582**	**0.008051**	**1.559392**	**1.122639–2.166059**
pN						
N0 vs. N1–N2	**0.000564**	**1.621062**	**1.231833–2.133276**	0.163298	1.285130	0.903181–1.828603
p63						
≤25 vs. >25%	0.097096	0.796109	0.608116–1.042217			
TTF-1						
≤25 vs. >25%	0.936561	0.989066	0.754490–1.296575			
Ki-67						
≤25 vs. >25%	0.968136	0.994309	0.751467–1.315628			
Cytoplasmic						
zyxin levels						
in cancer cells						
High vs. Low	0.506478	1.095422	0.837153–1.433368			
Nuclear						
zyxin levels						
in cancer cells						
Low vs. High	0.649726	1.064851	0.811928–1.396562			
**B**						
		**Overall Survival**				
		**SCC**				
**Univariate**				**Multivariate**		
**Clinical Feature**	***p* Value**	**HR**	**Confidence Interval 95% (HR)**	***p* Value**	**HR**	**Confidence Interval 95% (HR)**
Age						
≤64 vs. >64	0.083694	1.456415	0.951168–2.230043			
Sex						
Female vs. male	0.121189	1.595476	0.883699–2.880554			
Smoking						
No vs. Yes	0.748266	1.159491	0.469650–2.862597			
Living in						
urban areas						
No vs. Yes	0.502217	1.266994	0.634712–2.529139			
Clinical stage						
I–II vs. III–IV	**0.001125**	**2.128965**	**1.351164–3.354511**	0.247973	1.390731	0.794747–2.433645
Histological grade						
G1–G2 vs. G3	**0.000091**	**2.997641**	**1.729638–5.195220**	**0.002834**	**2.414826**	**1.353633–4.307953**
pT						
pT1-pT2 vs.						
pT3-pT4	**0.000534**	**2.148663**	**1.393743–3.312486**	**0.049517**	**1.682302**	**1.001099–2.827031**
pN						
N0 vs. N1–N2	0.908460	1.026365	0.658646–1.599380			
p63						
≤25 vs. >25%	0.365148	0.767183	0.432303–1.361474			
TTF-1						
≤25 vs. >25%	0.546339	1.199180	0.664646–2.163608			
Ki-67						
≤25 vs. >25%	0.836690	0.956480	0.626516–1.460224			
Cytoplasmic						
zyxin levels						
in cancer cells						
High vs. Low	0.603775	1.119886	0.730239–1.717444			
Nuclear						
zyxin levels						
in cancer cells						
Low vs. High	0.823766	0.953000	0.623872–1.455763			
**C**						
		**Overall Survival**				
		**AC**				
**Univariate**				**Multivariate**		
**Clinical Feature**	***p* Value**	**HR**	**Confidence Interval 95% (HR)**	***p* Value**	**HR**	**Confidence Interval 95% (HR)**
Age						
≤61 vs. >61	0.439639	1.170082	0.785611–1.742711			
Sex						
Female vs. male	**0.000411**	**2.195089**	**1.419128–3.395335**	**0.000119**	**2.371420**	**1.527428–3.681766**
Smoking						
No vs. Yes	0.331944	1.270446	0.783337–2.060456			
Living in						
urban areas						
No vs. Yes	**0.029915**	**2.244604**	**1.081842–4.657101**	**0.029913**	**2.314151**	**1.085072–4.935430**
Clinical stage						
I–II vs. III–IV	**0.000000**	**2.922240**	**1.945881–4.388492**	0.364952	1.311295	0.729573–2.356852
Histological grade						
G1–G2 vs. G3	0.880118	1.037945	0.639707–1.684099			
pT						
pT1-pT2 vs.						
pT3-pT4	**0.000001**	**2.846085**	**1.873553–4.323444**	**0.002122**	**2.191669**	**1.328609–3.615369**
pN						
N0 vs. N1–N2	**0.000000**	**2.883628**	**1.922023–4.326332**	**0.001392**	**2.317220**	**1.384130–3.879337**
p63						
≤25 vs. >25%	0.907950	1.032147	0.603679–1.764726			
TTF-1						
≤25 vs. >25%	0.133316	0.720412	0.469498–1.105423			
Ki-67						
≤25 vs. >25%	0.894629	1.033806	0.632080–1.690855			
Cytoplasmic						
zyxin levels						
in cancer cells						
High vs. Low	0.758237	1.064633	0.714493–1.586360			
Nuclear						
zyxin levels						
in cancer cells						
Low vs. High	0.246701	1.271330	0.846950–1.908353			

## Data Availability

The protocols and datasets will be made available to other researchers on reasonable request. For protocols or datasets, contact aleksandra.partynska@umw.edu.pl.

## Supplementary Materials

The following supporting information can be downloaded at: https://www.mdpi.com/article/10.3390/biom12060827/s1, Figure S1: Representative IHC images of ZYX expression in different subtypes of lung adenocarcinoma. Magnification x200.
