# Supplementary material for: Expression of Zyxin in Non-Small Cell Lung Cancer—A Preliminary Study
Source: Biomolecules. 2022 Jun 13;12(6):827. doi: 10.3390/biom12060827 (PMC9221212; doi:10.3390/biom12060827)
Supplement: Supplementary file 1 [file biomolecules-12-00827-s001.zip › SUPPLF~1.pdf]

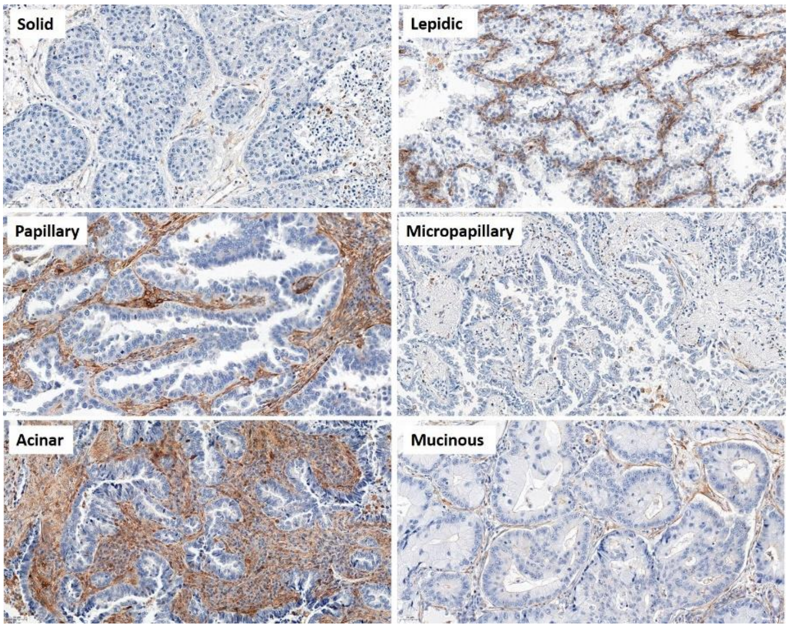

Supplementary Figure 1. Representative IHC images of ZYX expression in different subtypes of lung adenocarcinoma. Magnification x200.
